# Genotyping and spatial analysis of pulmonary tuberculosis and diabetes cases in the state of Veracruz, Mexico

**DOI:** 10.1371/journal.pone.0193911

**Published:** 2018-03-13

**Authors:** Francles Blanco-Guillot, M. Lucía Castañeda-Cediel, Pablo Cruz-Hervert, Leticia Ferreyra-Reyes, Guadalupe Delgado-Sánchez, Elizabeth Ferreira-Guerrero, Rogelio Montero-Campos, Miriam Bobadilla-del-Valle, Rosa Areli Martínez-Gamboa, Pedro Torres-González, Norma Téllez-Vazquez, Sergio Canizales-Quintero, Mercedes Yanes-Lane, Norma Mongua-Rodríguez, Alfredo Ponce-de-León, José Sifuentes-Osornio, Lourdes García-García

**Affiliations:** 1 Doctorado en Ciencias en Enfermedades Infecciosas, Centro de Investigación sobre Enfermedades Infecciosas, Instituto Nacional de Salud Pública, Cuernavaca, México; 2 Doctorado en Geografía, Universidad Nacional Autónoma de México, Ciudad de México, México; 3 Centro de Investigación sobre Enfermedades Infecciosas, Instituto Nacional de Salud Pública, Cuernavaca, México; 4 Laboratorio de Microbiología, Instituto Nacional de Ciencias Médicas y de Nutrición “Salvador Zubirán”, Ciudad de México, México; 5 Facultad de Medicina, Universidad Autónoma de San Luis Potosí, San Luis Potosí, México; 6 Maestría en Ciencias Médicas con énfasis en Epidemiología, Facultad de Medicina, Universidad Nacional Autónoma de México, Ciudad de México, México; 7 Dirección Médica, Instituto Nacional de Ciencias Médicas y de Nutrición “Salvador Zubirán”, Ciudad de México, México; Johns Hopkins University Bloomberg School of Public Health, UNITED STATES

## Abstract

**Background:**

Genotyping and georeferencing in tuberculosis (TB) have been used to characterize the distribution of the disease and occurrence of transmission within specific groups and communities.

**Objective:**

The objective of this study was to test the hypothesis that diabetes mellitus (DM) and pulmonary TB may occur in spatial and molecular aggregations.

**Material and methods:**

Retrospective cohort study of patients with pulmonary TB. The study area included 12 municipalities in the Sanitary Jurisdiction of Orizaba, Veracruz, México. Patients with acid-fast bacilli in sputum smears and/or *Mycobacterium tuberculosis* in sputum cultures were recruited from 1995 to 2010. Clinical (standardized questionnaire, physical examination, chest X-ray, blood glucose test and HIV test), microbiological, epidemiological, and molecular evaluations were carried out. Patients were considered “genotype-clustered” if two or more isolates from different patients were identified within 12 months of each other and had six or more IS6110 bands in an identical pattern, or < 6 bands with identical IS6110 RFLP patterns and spoligotype with the same spacer oligonucleotides. Residential and health care centers addresses were georeferenced. We used a Jeep hand GPS. The coordinates were transferred from the GPS files to ArcGIS using ArcMap 9.3. We evaluated global spatial aggregation of patients in IS6110-RFLP/ spoligotype clusters using global Moran´s I. Since global distribution was not random, we evaluated “hotspots” using Getis-Ord Gi* statistic. Using bivariate and multivariate analysis we analyzed sociodemographic, behavioral, clinic and bacteriological conditions associated with “hotspots”. We used STATA® v13.1 for all statistical analysis.

**Results:**

From 1995 to 2010, 1,370 patients >20 years were diagnosed with pulmonary TB; 33% had DM. The proportion of isolates that were genotyped was 80.7% (n = 1105), of which 31% (n = 342) were grouped in 91 genotype clusters with 2 to 23 patients each; 65.9% of total clusters were small (2 members) involving 35.08% of patients. Twenty three (22.7) percent of cases were classified as recent transmission. Moran`s I indicated that distribution of patients in IS6110-RFLP/spoligotype clusters was not random (Moran`s I = 0.035468, Z value = 7.0, p = 0.00). Local spatial analysis showed statistically significant spatial aggregation of patients in IS6110-RFLP/spoligotype clusters identifying “hotspots” and “coldspots”. GI* statistic showed that the hotspot for spatial clustering was located in Camerino Z. Mendoza municipality; 14.6% (50/342) of patients in genotype clusters were located in a hotspot; of these, 60% (30/50) lived with DM. Using logistic regression the statistically significant variables associated with hotspots were: DM [adjusted Odds Ratio (aOR) 7.04, 95% Confidence interval (CI) 3.03–16.38] and attending the health center in Camerino Z. Mendoza (aOR18.04, 95% CI 7.35–44.28).

**Conclusions:**

The combination of molecular and epidemiological information with geospatial data allowed us to identify the concurrence of molecular clustering and spatial aggregation of patients with DM and TB. This information may be highly useful for TB control programs.

## Introduction

Tuberculosis (TB) remains one of the main causes of morbidity and mortality in low- and medium-income countries, where the number of individuals with diabetes mellitus (DM) is rapidly increasing [1, 2]. In 2017, TB incidence rate in Mexico was 17 per 100,000 inhabitants indicating that the disease continues to represent a public health problem; while DM prevalence of 9.17% among individuals older than 20 years of age ranks sixth among adults worldwide [3, 4]. The convergence of both diseases in Mexico has led the International Diabetes Federation (IDF) to conclude that more than 10% of TB patients can be attributed to DM [5].

Many studies have explored the association between DM and TB, including a recent systematic review demonstrating that the risk of TB among people with DM triples that of people without DM [6]. Moreover, available evidence indicates that DM comorbidity worsens the clinical outcomes of TB patients [7, 8].

Genotyping of *Mycobacterium tuberculosis* together with conventional epidemiological methods has contributed to the characterization of strains [9–11] and has broadened the understanding of dynamics of transmission of TB in different places and specific populations [12–14]. Current evidence suggests that in addition to individual and social risk factors for genetic grouping of TB cases [12, 15–19], several ecologic, geographic, climatic and socioeconomic factors also have a critical impact on TB prevalence [20–22].

Geographical information systems (GIS) have become important tools in research and planning of TB programs [23–25]. GIS can be used to map rates of disease, define populations at risk, identify outbreaks and map social and environmental risk factors [23, 26]. Spatial aggregation of TB patients has been shown to occur both in high and middle and low-income settings [24, 26–28].

Several research groups have combined genotypes with geospatial data to characterize TB distribution and transmission within specific groups [28–32]. To our knowledge, the hypothesis that recent transmission and spatial aggregation occurs among TB patients with DM has not been previously tested [33, 34], although clinical manifestations among patients with TB and DM such as delayed sputum and culture conversion [8, 35, 36], higher likelihood of pulmonary (versus extra-pulmonary) forms [37] and cavitation [8] due to dysfunctional innate and acquired immune system indicate that patients with DM might have an important role in TB transmission. In addition, patients with DM and hyperglycemia have a greater risk of infection and disease [38], which would increase their likelihood of participating in chains of transmission. Identification of high-risk subpopulations and areas where TB transmission is occurring would be useful to focus prevention and control strategies where they might be more effective.

Since 1995, we have been conducting a population-based study of pulmonary TB in Southern Mexico where almost one-third of TB patients have been previously diagnosed with DM [39]. The purpose of the present study was to test the hypothesis that DM and pulmonary TB may occur in spatial and molecular aggregations. If confirmed, spatially heterogeneous interventions may be considered to interrupt transmission in specific locations.[40].

## Material and methods

### Study population and recruitment

We carried out a retrospective analysis of a prospectively recruited cohort of pulmonary TB patients. The methods of selection, recruitment and follow up have been previously described [8, 39]. The study area includes 12 municipalities of the Sanitary Jurisdiction of Orizaba in the State of Veracruz, Mexico. The site has an area of 618.11km2 with 413,223 inhabitants, of which 26.3% live in rural communities [8]. TB incidence in Veracruz ranked among the ten highest in Mexico in 2015 with 27.3 cases per 100,000 inhabitants [3]. In 2012, state prevalence of DM among individuals 20 years and older ranked third in Veracruz with 10.7%. [4]. Health coverage for the study area is provided by public and private institutions. Public institutions include the Ministry of Health (SSA) for the uninsured population, Seguro Popular which is a program of comprehensive national health insurance for the previously uninsured; the Mexican Social Security Institute (IMSS) for the employed population; the State Workers Social Security Services Institute (ISSSTE) for government employees and Petróleos Mexicanos (Pemex) for oil workers. Because antituberculosis medications are available free of charge through the public health institutions, private physicians provide care for relatively few tuberculosis patients. Primary health care is provided through 48 rural and urban health centers.

Briefly, between March 1995 and April 2010, ongoing recruitment was performed by community health workers who were trained to identify patients with cough persisting more than two weeks among members of the community. Additionally, shelters, jails, orphanages, and self-support groups for users of alcohol or illegal drugs and patients living with diabetes were visited periodically to explain the purpose of the study and identify patients with respiratory symptoms. Study personnel invited participation of patients older than 20 years of age with acid-fast bacilli (AFB) in sputum smear or *Mycobacterium tuberculosis* in sputum culture. Each consenting patient completed a clinical exam (including a standardized questionnaire, physical examination, chest x-ray, blood glucose and HIV test) and provided sputum samples for microbiological and molecular tests. Subsequent episodes of TB in the same patient were also documented. Chest x-rays were independently evaluated by certified radiologists. Trained personnel administered previously validated standardized questionnaires. Sputum cultures were conducted from 1995 to 1999 in samples with acid-fast bacilli (AFB), from 2000 to 2005 in all sputum samples, and from 2005 to 2010 in previously treated TB patients, household contacts of patients with drug resistance and patients with DM.

Drug susceptibility test results were sent to treating physicians. Patients received treatment in the local health centers. The majority of patients received treatment in a single center.

### Definitions

Diabetes mellitus: Patients were considered to have DM if they had received a previous diagnosis from a physician, had been prescribed oral hypoglycemic medication or insulin before TB diagnosis or had resulted with 200 mg/dl or higher of glucose in a random test when diagnosed with TB.

Voluntary HIV testing and counseling was offered to all participants. Results were informed to the patient. In case of positive results, he/she was referred to receive appropriate treatment. Testing for HIV was done as per the Mexican HIV Prevention and Control Program using two different tests [41]. All positive results were confirmed by Western blot. Previous HIV diagnosis was also considered.

Rural residence (towns with less than 2,500) and homelessness were defined as in the Population and Household Census [42]. Usage of alcohol (> 10 drinks per week), smoking (> 10 cigarettes per week), usage of illegal drugs, (marijuana, cocaine and its derivatives, heroin, methamphetamines, hallucinogens, inhalants and other drugs) were defined as in the National Survey of Addictions (NSA) (SSA, 2005). This information was obtained through self-report.

Body mass index was calculated as weight in kilograms divided by the square of the height in meters (kg/m^2^). To evaluate health care access, we assessed the distance to the nearest health center and the time elapsed between the onset of symptoms and the beginning of treatment.

### Mycobacteriology and genotyping

Ziehl Neelsen stain, mycobacteria culture, species identification and drug susceptibility tests (DST) were conducted using standardized procedures [43]. Isolates were genotyped and compared using IS6110-based restriction fragment-length polymorphisms (RFLP) and spacer oligonucleotide typing (spoligotyping). If the isolate’s IS6110 RFLP patterns had fewer than 6 bands [44] patients were considered “genotype-clustered” if two or more isolates from different patients were identified within 12 months of each other and had six or more IS6110 bands in an identical hybridization banding pattern, or < 6 bands with identical IS6110 RFLP patterns and a spoligotype with the same spacer oligonucleotides. [45]. We used a single isolate for each episode of TB. We used the “n minus one” method to estimate ongoing transmission [46]. Microbiology and molecular tests were conducted at the Mycobacteriology Laboratory of the Instituto Nacional de Ciencias Médicas y de Nutrición Salvador Zubirán.

### Spatial analysis

We georeferenced all patient’s residential addresses at the time of diagnosis. We also georeferenced all health centers in the study area. We used a Jeep hand GPS. The coordinates were transferred from the GPS files to ArcGIS using ArcMap 9.3. The statistical analysis was done using ArcMap 10.3.

We analyzed spatial aggregation of genotype-clustered patients (defined as described above: IS6110-RFLP/spoligotype clustered TB patients identified within 12 months of each other) and patients harboring unique patterns using global Moran`s I, which is a measure of spatial autocorrelation [47]. This tool tested for the occurrence of spatial aggregated, dispersed, or random patient distribution. Moran`s I values vary from -1 to 1, with 1 being the maximum positive association and -1 for the maximum negative association. There was no correlation when the value was 0; a higher positive value indicated a stronger spatial correlation; negative values meant weaker spatial correlation. Z-score was used to evaluate the significance of the estimation of Moran`s I. We considered TB cases to be spatially and statistically aggregated when Moran`s I was >0 and Z-score is ≥1,96 [48].

#### Hotspot analysis

Since the results of Moran´s I showed a non-random distribution, we investigated whether patients forming genotype clusters showed local indicators of spatial aggregation (LISA or hotspots) using the Getis-Ord Gi* tool [49, 50]. This tool identifies spatial aggregations which are statistically significant with high scores (hotspots) and low scores (coldspots) and confidence level bins (Gi_Bin). Features in the +/-3; +/-2 and +/-1 bins were statistically significant at the 99%, 95%, and 90% confidence level respectively [51]. Spatial aggregation for features with 0 for the Gi_Bin field was not statistically significant. To determine spatial aggregation using this tool, we used the identifier of each IS6110-RFLP/spoligotype molecular cluster as “Analysis Field.” Therefore, hotspots refer to spatial aggregations of patients in the same IS6110-RFLP/spoligotype molecular cluster. We also analyzed data filtering the study population (in molecular clusters) according to whether patients had been diagnosed with DM or not. It is important to note that this method does not allow analysis of patients with unique patterns since according to our genotype cluster definition (two or more patients sharing identical patterns), patients with unique patterns were not assigned an identifier of IS6110-RFLP/spoligotype molecular cluster. We chose to use a default distance equivalent to the minimum distance to ensure that every genotype-clustered patient had at least one neighbor of the same molecular cluster. The default neighborhood search threshold was 3774.6351 meters.

### Statistical analysis

We used bivariate analyses to test for differences in sociodemographic, behavioral, clinical and bacteriological characteristics between patients who were fingerprinted with those that were not; patients in IS6110-RFLP/spoligotype clusters with those harboring unique fingerprints; and genotype-clustered patients in hotspots with genotype-clustered patients not in hotspots. Among patients in genotype-clusters, we used logistic regression to assess variables associated to hotspots. Variables with p 0.20 in the bivariate analysis and biological plausibility were included in multivariate models. We estimated the odds ratio (OR) and 95% CI and identified the covariates that were independently associated with each outcome. We built five models: using overall population and the following subgroups (patients with and without diabetes, and patients diagnosed between 1995 and 1999 and patients diagnosed between 2000 and 2010). In the subgroup analyses, we used the same covariates as in the overall model since we wanted to investigate if DM diagnosis and attendance at the urban health center of Camerino Z. Mendoza continued being associated with hotspots in each of the subgroups. All data analysis was performed using STATA 13.1.

### Ethical approval

Participants provided written informed consent to participate in this study. Ethical approval was obtained from the Ethical Commission of the *Instituto Nacional de Salud Pública* (approval number = 527). All participants were referred to health facilities to receive treatment in accordance with the stipulations of the National Program for the Prevention and Control of TB.

## Results

**Fig 1:**
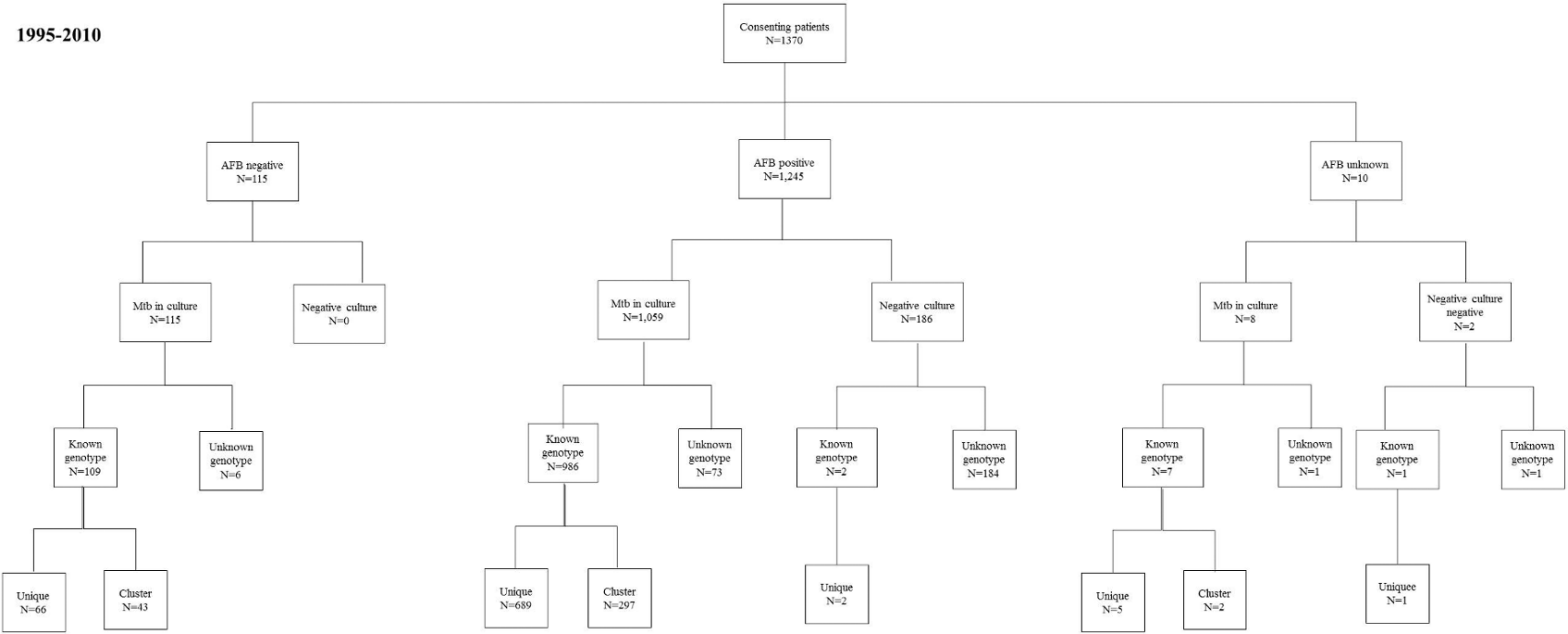
Number of individuals at each stage of the study (1995–2010).

Between 1995 and 2010, 1370 patients older than 20 years were diagnosed with pulmonary TB; of these, 33% had DM. Eighty percent of M tuberculosis strains were genotyped (80.66%, (1105/1370); 31% of genotyped isolates (342/1105) formed IS6110-RFLP/spoligotype clusters. The flow diagram shows the different numbers of individuals at each stage of the study (Fig 1).The proportion of isolates that were genotyped according to the three different methods of diagnosis of patients used during the study period (cultures on all AFB positive, cultures on all samples and cultures on previously treated TB patients, household contacts of patients with drug resistance and patients with DM) was 76.05% (327/430), 1995 to 1999; 83.69% (508/607), 2000 to 2005; and 81.08% (270/333), 2005 to 2010 (S1–S3 Figs).

Patients without fingerprints were less likely to have been diagnosed from 2000 to 2010 (61%, (162/265) *vs*. 70%, (778/1105) p = 0.003), use alcohol (38%, (101/265) *vs*. 45%,(494/1103) p = 0.049), have DM (26%, (70/265) *vs*. 34%, 380/1105) p = 0.013), harbor a pan-susceptible strain (66%, (42/64) *vs*. 78% (739/945) p = 0.02), have fever (64%, (169/265) *vs*. 71%, (787/1102) p = 0.015) or cavitations in the chest X-ray (22% (46/213) *vs*. 44%, 411/942) *vs*. p = <0.001).

There were no differences regarding other demographic, socioeconomic, epidemiological or clinical variables (S1 Table). We compared characteristics of patients with and without fingerprints according to the three different methods of diagnosis of patients used during the study period and found some additional differences. Patients without fingerprints were less likely to have cavities in chest X-ray, and more likely to have earthen floors and live in rural areas in the period 1995 to 1999; and they were older and less likely to smoke and use drugs in the period 2000 to 2005 (S2–S4 Tables).

Table 1 shows five categories of patients in IS6110-RFLP/spoligotype clusters according to the number of members of each genotype cluster. In total, 342 genotype-clustered patients were distributed in 91 IS6110-RFLP/spoligotype clusters involving 2 to 23 patients; 65.9% of molecular clusters were small (2 members) involving 35.08% of patients. The proportion of cases attributed to recent TB transmission in the cohort was estimated at 22.7%.

**Table 1:** Number of patients according to IS6110-RFLP/ spoligotype cluster size.

Category	Cluster size	Number of IS6110-RFLP/ spoligotype clusters	Total number of patients
A	2	60	120
B	3–5	17	63
C	6–10	9	71
D	11–20	3	44
E	21–23	2	44

Table 2 shows the comparison between patients in IS6110-RFLP/spoligotype clusters and patients with a unique pattern. Patients in IS6110-RFLP/spoligotype clusters were more likely to have been diagnosed after 2000 (77% (262/342) *vs*. 68% (516/763), p = 0.002), to be drug users (8% (26/342) *vs*. 4% (33/762), p = 0.025), to harbor pan-susceptible strains (82% (250/304) *vs*. 76% (489/641), p = 0.039) and less likely to harbor MDR strains (4% (11/304) *vs*. 8% (50/641), p = 0.015). DM did not show a statistically significant difference between both groups (32% (108/342) *vs*. 36 (272/763), p = 0.188).

**Table 2:** Comparison of sociodemographic and clinical characteristics between patients in IS6110-RFLP/ spoligotype clusters and patients with unique patterns.

Characteristics	Total	Unique fingerprint	Genotype- Clustered	p-value
	n/total (%)	n/total (%)	n/total (%)	
Male	659/1105 (60.0)	447/763 (59.0)	212/342 (62.0)	0.286^d^
Mean (SD) age (years)	45.5 (17.6)	46.1 (17.1)	44.0 (18.7)	0.071 ^f^
>6 years of formal schooling	767/1104 (69.0)	525/762 (69.0)	242/342 (71.0)	0.534 ^d^
Household with earthen floor	221/1105 (20.0)	147/763 (19.0)	74/342 (22.0)	0.362 ^d^
Rural residence	118/992 (12.0)	83/691 (12.0)	35/301 (12.0)	0.864 ^d^
Median (IQR) distance to nearest health center (meters)	696 (413–1034)	717 (412–1072)	657 (433–976)	0.241^e^
Diagnosis between 2000 and 2010 *vs*. 1995 to 1999	778/1105 (70.0)	516/763 (68.0)	262/342 (77.0)	0.002 ^d^
Access to Social Security	376/1105 (34.0)	267/763 (35.0)	109/342 (32.0)	0.311 ^d^
Mean (SD) body mass index	21.2 (5.3)	21.4 (5.7)	20.7 (4.1)	0.046^f^
Urban health center in Camerino Z. Mendoza	98/778 (13.0)	57/438 (13.0)	41/340 (12.0)	0.691 ^d^
>10 drinks per week	494/1103 (45.0)	326/761 (43.0)	168/342 (49.0)	0.052 ^d^
>10 cigarettes per week	261/1103 (24.0)	178/761 (23.0)	83/342 (24.0)	0.751 ^d^
Use of illegal drugs	59/1104 (5.0)	33/762 (4.0)	26/342 (8.0)	0.025 ^d^
Homelessness or residing in shelters	34/1102 (3.0)	21/761 (3.0)	13/341 (4.0)	0.35 ^d^
BCG^a^ scar	505/1100 (46.0)	346/760 (46.0)	159/340 (47.0)	0.703 ^d^
HIV^b^ infection	22/1070 (2.0)	15/737 (2.0)	7/333 (2.0)	0.943 ^d^
Median (IQR) time elapsed between onset of symptoms and treatment (days)	107 (62–192)	109 (65–197)	105 (62–180)	0.174^e^
New tuberculosis patients	908/1104 (82.0)	631/763 (83.0)	277/341 (81.0)	0.555 ^d^
Diabetes Mellitus	380/1105 (34.0)	272/763 (36.0)	108/342 (32.0)	0.188 ^d^
Number of bacilli per oil immersion field				
Smear negative/culture positive (paucibacillary)	109/1097 (10.0)	66/757 (9.0)	43/340 (13.0)	0.246 ^d^
10 to 99 AFB^c^ per 100 immersion fields	357/1097 (33.0)	251/757 (33.0)	106/340 (31.0)	0.246 ^d^
1 to 10 AFB^c^ per oil immersion field	332/1097 (30.0)	230/757 (30.0)	102/340 (30.0)	0.246 ^d^
More than 10 AFB^c^ per oil immersion field	299/1097 (27.0)	210/757 (28.0)	89/340 (26.0)	0.246 ^d^
Sensitive	739/945 (78.0)	489/641 (76.0)	250/304 (82.0)	0.039 ^d^
Multidrug resistant	61/945 (6.0)	50/641 (8.0)	11/304 (4.0)	0.015 ^d^
Fever	787/1102 (71.0)	555/762 (73.0)	232/340 (68.0)	0.119 ^d^
Hemoptysis	366/1101 (33.0)	246/759 (32.0)	120/342 (35.0)	0.383 ^d^
Cavities on chest x-ray	411/942 (44.0)	273/647 (42.0)	138/295 (47.0)	0.188 ^d^

^a^BCG: vaccine against Bacillus Calmette-Guérin

^b^HIV: human immunodeficiency virus

^c^AFB: acid fast bacilli

^d^ X^2^ test

^e^Kruskall Wallis test

^f^ Student's t-test.

SD, Standard deviation; IQR, Interquartile range.

### Spatial distribution and hotspot analysis

**Fig 2:**
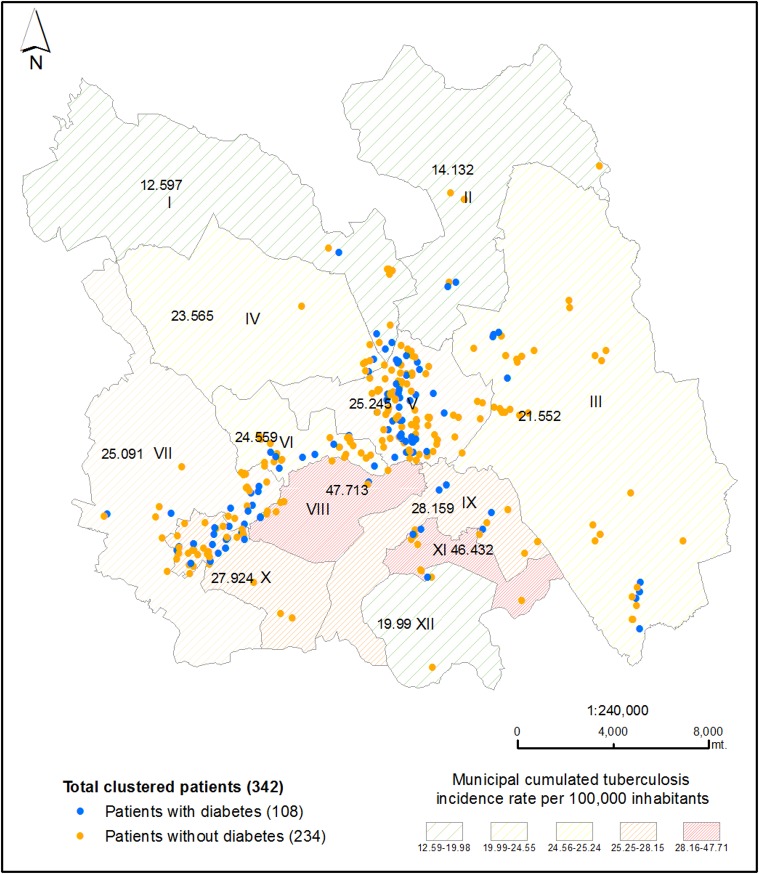
Geographical distribution of genotype-clustered patients in the study site. Yellow dots indicate genotype-clustered patients without DM. Blue dots indicate genotype-clustered patients with DM. The hashed pattern indicates TB rates in each municipality (the green spaces stripes indicate lower rates, the red coloring indicate higher rates). The number within each municipality shows the exact rate. Municipalities: Mariano Escobedo (I), Atzacan (II), Ixtaczoquitlán (III), Ixhuatlancillo (IV), Orizaba (V), Río Blanco (VI), Nogales (VII), Huiloapan (VIII), Rafael Delgado (IX), Camerino Z. Mendoza (X), Tlilapan (XI) and San Andrés Tenejapan (XII) [52].

The geospatial distribution of 342 IS6110-RFLP/spoligotype clustered patients is shown in Fig 2 including 108 patients with DM. Patients were concentrated around the Orizaba municipality and extending toward surrounding municipalities (Río Blanco, Nogales, and Camerino Z. Mendoza municipalities). Even though municipalities such as Huiloapan de Cuauhtemoc and Tlilapan had a higher incidence rate, we did not observe spatial aggregation in these localities.

Of the 91 genetic clusters, 20 (22%) had their first identified or "index" case located within the Mendoza or Nogales municipalities (10 each). Mendoza municipality concentrated 2 of 15 cases and 12 of 21 cases of the two largest molecular clusters (D and E).

Moran`s I for IS6110-RFLP/spoligotype clustered patients detected a global pattern of spatial autocorrelation (Moran`s I = 0.035468, Z value = 7.0, p = 0.00). That is, distribution of patients in IS6110-RFLP/ spoligotype clusters was not random.

**Fig 3:**
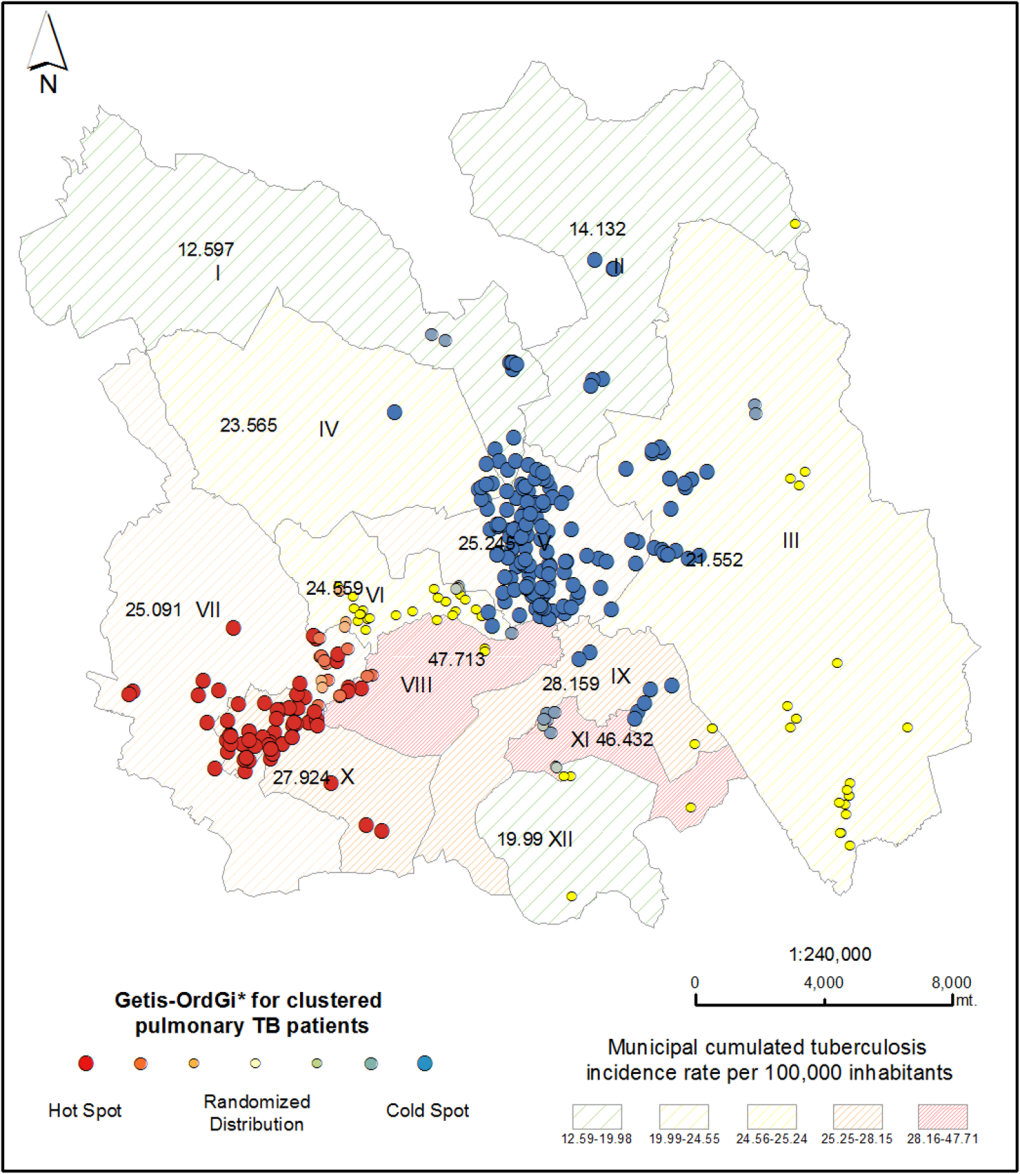
Local spatial analysis of genotype-clustered patients in the study area. The scale shows Z value scores: the higher the score of Z, the greater the tendency to spatial aggregation (hotspots), shown in red dots. For negative Z values that are statistically significant, the lower the score of Z, the greater the tendency to dispersion (cold spots) shown in blue dots. Random distribution (between -1.96 and 1.96) is shown in yellow. Almost fifteen percent (50/342) of patients had a Z value above 1.96 and were located in Camerino Z. Mendoza and Nogales municipalities compared to patients in the rest of the municipalities that had lower Z values. These values indicate that patients in genotype clusters were not randomly distributed but spatially aggregated. Municipalities identified by Roman numerals as in Fig 2 [52].

Local spatial analysis using Getis-Ord Gi* statistic showed statistically significant spatial aggregation of patients in IS6110-RFLP/ spoligotype clusters forming “hotspots” and “coldspots”. Of the patients in IS6110-RFLP/spoligotype clusters, 14.6% (50/342) were part of the hotspot in Camerino Z. Mendoza and Nogales municipalities, of these 60% (30/50) were patients with DM. Orizaba municipality was identified as a coldspot, that is, the distance between patients in IS6110-RFLP/ spoligotype clusters was larger, and the frequency of spatial aggregation was lower (Fig 3).

**Fig 4:**
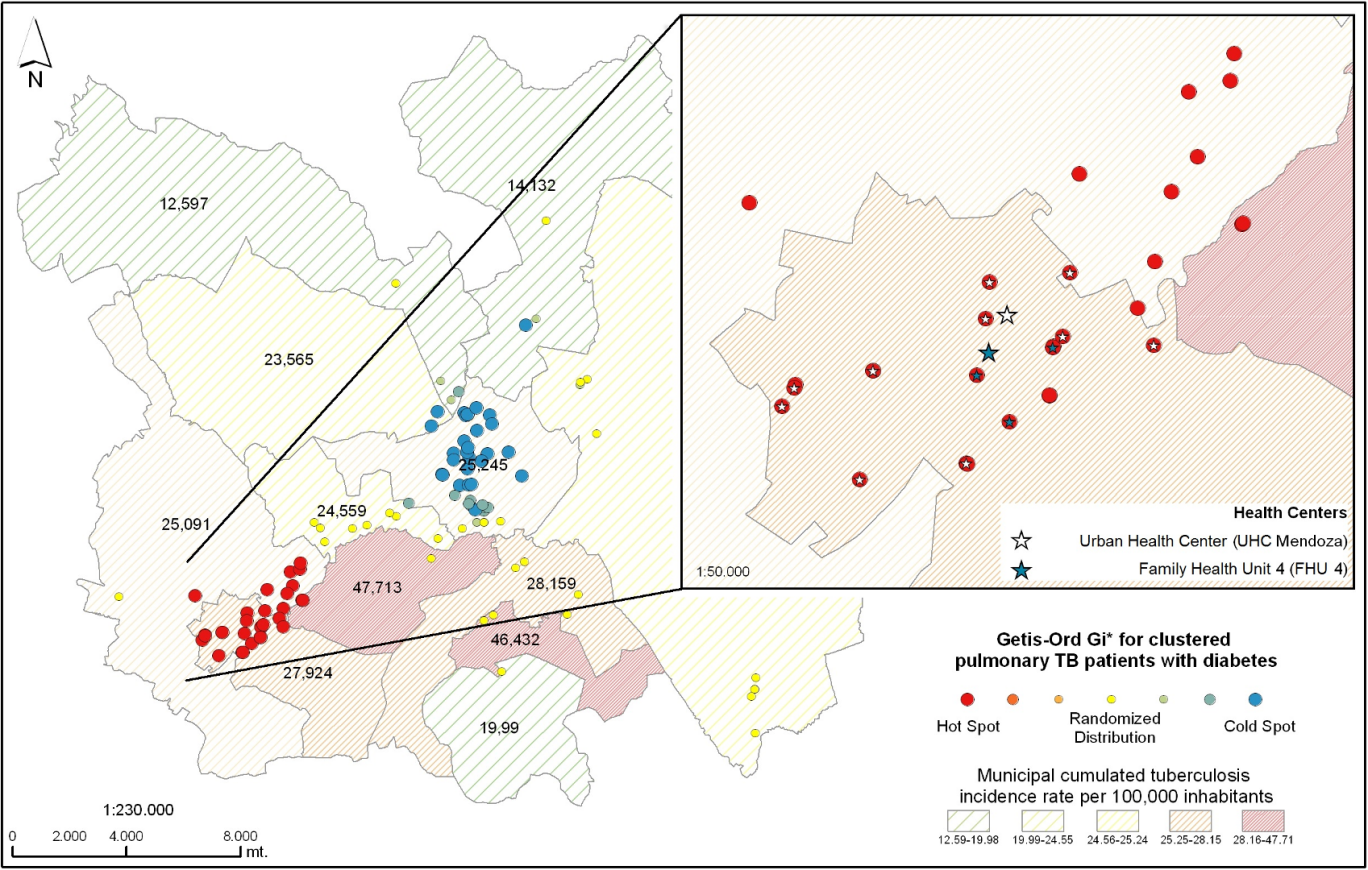
Local spatial analysis of genotype-clustered TB/DM patients in the study area. Getis-Ord Gi* showed a hotspot in Camerino Z. Mendoza and Nogales municipalities. 27% (29/108) of patients with DM had Z scores above 1.96 (red dots). In the other municipalities, Z scores are lower than1.96 which means there was random spatial distribution (yellow dots) or a coldspot (blue dots). Thirty-eight percent (41/108) of patients with diabetes had Z scores lower than -1.96 (blue dots), and 88% (36/41) were in the Orizaba municipality. The zoomed area shows the urban health center of Mendoza (white star) and the #4 IMSS family heath unit (blue star). The star in the center of each spatial aggregate indicates the health center where patients received clinical care; 38% (11/29) attended the urban health center of Mendoza and 10% (3/29) the #4 IMSS family health unit of the same municipality. The remaining 52% (15/29) either attended the health center in Nogales municipality or other health centers. Municipalities identified by Roman numerals as in Fig 2 [52].

Patients with both TB and DM showed statistically significant spatial aggregation in the Camerino Z. Mendoza and Nogales municipalities (Fig 4). We reviewed clinical files of patients in hotspots and found that the majority (38% (11/29), received clinical care in one urban health center in Mendoza (Fig 4).

**Fig 5:**
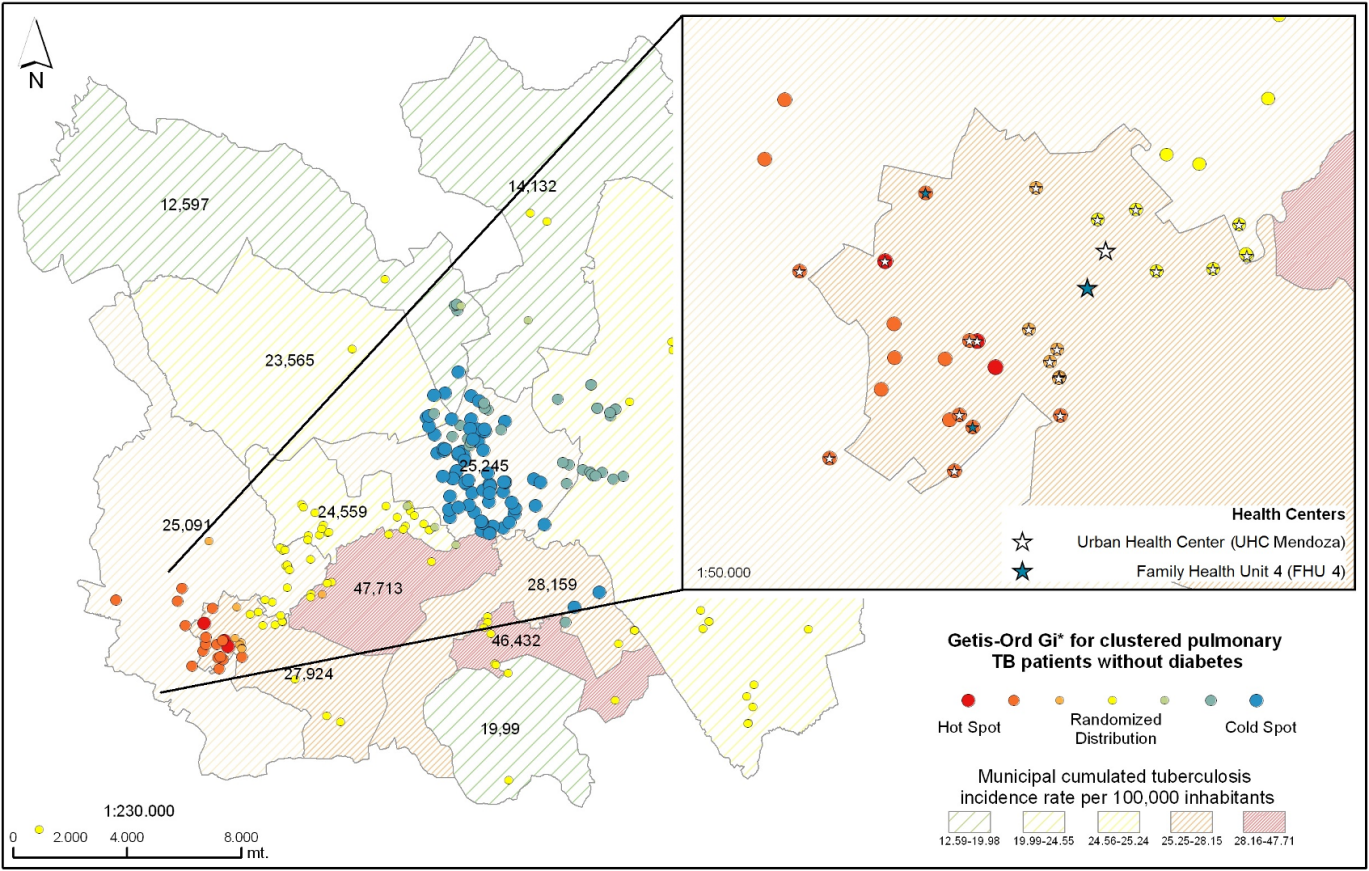
Local spatial analysis of genotype-clustered TB/non-DM patients in the study area. Z value scale as shown in Fig 2. Only 8.5% (20/234) of patients without DM had a Z score higher than 1.96 compared to patients of the rest of the municipalities that had lower Z scores. For the rest of patients, Z score values were lower than 1.96 indicating random spatial distribution (yellow dots) or coldspots (blue dots).The results of the hotspot are shown in the zoomed area; 40% (8/20) attended the urban health center in Mendoza, 10% (2/20) the #4 IMSS family health unit and 50% (10/20) other centers. Municipalities identified by Roman numerals as in Fig 2 [52].

The local spatial analysis of patients without DM in IS6110-RFLP/spoligotype clusters (234/342) identified a higher likelihood of patient concentration belonging to the same molecular cluster but without forming a hotspot in Camerino Z. Mendoza. This is shown in orange dots (Fig 4). When expanded, this area showed that patients mainly attended the urban health center of Mendoza (40% (8/20) (Fig 5).

Spatial autocorrelation of 763 genotypically unclustered patients including 272 patients with DM is shown in S4 Fig. Moran´s I revealed a pattern not significantly different than random. (Moran`s I = -0.001106, Z value = 0.085084, p = 0.9).

Table 3 shows the comparison between patients in IS6110-RFLP/ spoligotype clusters belonging to a hotspot with those not belonging to a hotspot. The main significant differences were distance to the health center (958mt, (486–1462) *vs*. 627mt (410–918), p = 0.005), receiving health care at the urban health center of Camerino Z. Mendoza (49%, (24/49) *vs*. 6%, (17/291), p = <0.001) and having been diagnosed with DM (60%, (30/50) *vs*. 27%, (78/292), p = <0.001).

**Table 3:** Comparison of sociodemographic and clinical characteristics of genotype-clustered patients according to belonging to a hotspot or not.

Characteristics	Total	Not in a hotspot	In hotspot	p-value
	n/total (%)	n/total (%)	n/total (%)	
Male	212/342 (62.0)	185/292 (63.0)	27/50 (54.0)	0.208 ^d^
Mean (SD) age (years)	44.0 (18.6)	43.4 (18.8)	47.9 (17.3)	0.113 ^f^
>6 years of formal schooling	242/342 (71.0)	205/292 (70.0)	37/50 (74.0)	0.586 ^d^
Household with earthen floor	74/342 (22.0)	64/292 (22.0)	10/50 (20.0)	0.761 ^d^
Rural residence	35/301 (12.0)	30/260 (12.0)	5/41 (12.0)	0.903 ^d^
Median (IQR) distance to nearest health center (meters)	657 (433–976)	627 (410–918)	958 (486–1462)	0.005^e^
Diagnosis between 2000 and 2010	262/342 (77.0)	222/292 (76.0)	40/50 (80.0)	0.54 ^d^
Access to Social Security	109/342 (32.0)	94/292 (32.0)	15/50 (30.0)	0.759 ^d^
Mean (SD) body mass index	20.7 (4.1)	20.7 (3.9)	21.0 (4.8)	0.545 ^f^
Urban health center of Camerino Z. Mendoza	41/340 (12.0)	17/291 (6.0)	24/49 (49.0)	<0.001 ^d^
>10 drinks per week	168/342 (49.0)	144/292 (49.0)	24/50 (48.0)	0.864 ^d^
>10 cigarettes per week	83/342 (24.0)	74/292 (25.0)	9/50 (18.0)	0.263 ^d^
Use of illegal drugs	26/342 (8.0)	25/292 (9.0)	1/50 (2.0)	0.106 ^d^
Homelessness or residing in shelters	13/341 (4.0)	13/292 (4.0)	0/49 (0.0)	0.132 ^d^
BCG^a^ scar	159/340 (47.0)	141/291 (48.0)	18/49 (37.0)	0.128 ^d^
HIV^b^ infection^b^	7/333 (2.0)	6/285 (2.0)	1/48 (2.0)	0.992 ^d^
Median (IQR) time elapsed between onset of symptoms and treatment (days)	105 (62–180)	100 (61–180)	108 (64–193)	0.455^e^
New tuberculosis patients	277/341 (81.0)	238/292 (82.0)	39/49 (80.0)	0.751 ^d^
Diabetes Mellitus	108/342 (32.0)	78/292 (27.0)	30/50 (60.0)	<0.001 ^d^
AFB in sputum				
Smear negative/culture positive (paucibacillary)	43/340 (13.0)	38/290 (13.0)	5/50 (10.0)	0.746 ^d^
10 to 99 AFB^c^ per 100 immersion fields	106/340 (31.0)	88/290 (30.0)	18/50 (36.0)	
1 to 10 AFB^c^ per oil immersion field	102/340 (30.0)	86/290 (30.0)	16/50 (32.0)	
More than 10 AFB^c^ per oil immersion field	89/340 (26.0)	78/290 (27.0)	11/50 (22.0)	
Drug susceptible	250/304 (82.0)	212/256 (83.0)	38/48 (79.0)	0.544 ^d^
Multidrug resistant	11/304 (4.0)	11/256 (4.0)	0/48 (0.0)	0.144 ^d^
Fever	232/340 (68.0)	197/290 (68.0)	35/50 (70.0)	0.772 ^d^
Haemoptysis	120/342 (35.0)	98/292 (34.0)	22/50 (44.0)	0.153 ^d^
Cavities on chest x-ray	138/295 (47.0)	112/252 (44.0)	26/43 (60.0)	0.052 ^d^

^a^BCG: vaccine against Bacillus Calmette-Guérin

^b^HIV: human immunodeficiency virus

^c^AFB: acid fast bacilli

^d^ X^2^ test

^e^Kruskall Wallis test

^f^ Student's t-test.

SD, Standard deviation; IQR, Interquartile range.

Multivariate analyses adjusted by sociodemographic and clinical variables (Table 4) showed that DM patients (OR 7.047, 95% CI 3.03, 16.38, p<0.001) and those receiving clinical care in the urban health center of Camerino Z. Mendoza (OR 18.04, 95% CI 7.35, 44.28, p<0.001) had a higher probability of belonging to hotspots.

**Table 4:** Multivariate analysis for characteristics of genotype-clustered patients associated with belonging to a hotspot.

Characteristic	Total	Diagnosis of DM		Study period	
		With	Without	1995 to 1999	2000 to 2010
	aOR^a^	aOR^a^	aOR^a^	aOR^a^	aOR^a^
	(95%CI^b^)	(95%CI^b^)	(95%CI^b^)	(95%CI^b^)	(95%CI^b^)
n	339	92	233	260	57
Female	1.098	0.783	1.604	1.202	0.811
	[0.52,2.33]	[0.21,2.95]	[0.56,4.63]	[0.52,2.79]	[0.09,7.40]
Age (years)	1	0.982	1.006	1	1.011
	[0.98,1.02]	[0.93,1.03]	[0.98,1.03]	[0.98,1.03]	[0.93,1.10]
Diabetes mellitus	7.047***	—-	—-	7.112***	12.85*
	[3.03,16.38]	—-	—-	[2.70,18.72]	[1.19,138.65]
Household with earthen floor	0.894	0.413	1.242	0.523	6.544
	[0.34,2.34]	[0.05,3.68]	[0.40,3.86]	[0.17,1.60]	[0.59,72.16]
Without Access to Social Security	1.124	1.19	0.828	0.878	1
	[0.49,2.56]	[0.35,3.99]	[0.25,2.75]	[0.35,2.19]	[1.00,1.00]
Distance to nearest health center (meters)	1.538	1.24	1.587	2.380**	1.115
	[0.96,2.47]	[0.43,3.59]	[0.97,2.59]	[1.28,4.42]	[0.72,1.73]
Urban health center of Camerino Z. Mendoza	18.04***	—— c	8.012***	23.35***	17.08*
	[7.35,44.28]	—-	[2.63,24.44]	[8.13,67.10]	[1.55,188.77]
Diagnosis between 2000 and 2010	1.74	2.136	1.912	—-	—-
	[0.65,4.69]	[0.43,10.66]	[0.43,8.50]	—-	—-

^a^OR = Odds ratio;

^b^CI95% = Confidence interval 95%

p Value =

* p<0.05

** p<0.01

*** p<0.001.

^c^ Omitted all DM patients attend to Urban health center of Camerino Z. Mendoza.

When we stratified by DM diagnosis, we found that attendance at the urban health center of Camerino Mendoza was associated with belonging to a hotspot among DM patients (all DM patients attended the urban health center of Camerino Z. Mendoza) and non-DM patients (OR 8.012 (95% CI 2.63–24.44), p<0.001) (Table 4).

In the models in which we stratified in two periods according to date of recruitment, results showed that DM (OR 7.112 (95% CI 2.70–18.72), p<0.001), living near the health center (OR 2.38 (95% CI 1.28–4.42) p<0.01) and attending the urban health center of Camerino Mendoza (OR 23.35 (95% CI 8.13–67.10), p<0.001) were associated with belonging to hotspots among patients recruited at earlier dates (1995 to 1999). Among patients recruited after 2000, belonging to hotspots was associated with DM (OR 12.85 (95% CI 1.19–138.65), p<0.05) and attending clinics in Camerino Z. Mendoza municipalities (OR 17.08 (95% CI 1.55–188.77), p<0.05) (Table 4).

## Discussion

The combination of molecular and epidemiological information with geospatial data allowed us to identify the occurrence of molecular clustering and spatial aggregations of patients with DM and TB. These results indicate the need to conduct prospective research to determine if health care might be facilitating transmission among DM patients and to implement the collaborative framework recommended by WHO and the Union broadly to prevent and control TB among patients with DM. [53]

Most of our patients were diagnosed with DM before or at the same time of their diagnosis with TB. Therefore, it is biologically plausible that immunological dysfunction associated with DM might favor their participation in transmission chains when exposed to infectious patients. Our hypothesis that health centers might be the source of TB infection for patients with DM is based on the finding that patients with TB and DM carrying the same genotype were geospatially aggregated around the health center where they received health care. The finding of localized transmission did not correspond to the most densely populated areas; on examination of population densities of the study area, we found that Camerino Mendoza Municipality ranked third after Orizaba and Rio Blanco municipalities [52]. Moreover, spatial autocorrelation of genotypically unclustered patients revealed a pattern not significantly different than random. The finding that transmission might be occurring in localized areas can have several alternative explanations. We limited geocoding to the patient´s residential address and to the health centers where each patient had received health care. Therefore, we did not consider other settings where transmission might have occurred such as transportation routes, workplaces or social gatherings in the same “hotspot” areas. Patients with the hotspot genotypes occurring outside the hotspot could be seeding larger areas. Therefore, timely detection of hotspots could prevent further dissemination.

As occurring for the rest of Mexico, and other countries worldwide [54–56], the frequency of DM among TB patients in the study was high (32.84%). If our hypothesis that patients with TB and DM occur in spatial and molecular aggregations proves to be correct, control efforts could be implemented in specific high-risk areas. Patients with DM have not been specifically studied, although clinical manifestations among patients with TB and DM indicate that patients with DM might have an important role in TB transmission. Moreover, patients with DM have been described as index and secondary cases in TB outbreaks [57, 58]. Usage of molecular epidemiologic techniques has previously allowed us to show that increased risk of TB among patients with DM is due to both reactivation and recently transmitted infection [39]. More recently, we demonstrated that patients with DM were reinfected with a different strain as the one that caused the initial episode in one-fifth of the cases [8]. We conjectured that exogenous TB reinfection in DM patients might be due to TB transmission associated with health care occurring as a result of DM patients attending clinics where there is a high prevalence of diagnosed and undiagnosed TB, as has been described for HIV infected patients [59].

The low percentage of clustering and attributable recent transmission found in this study could result from decreasing morbidity or effective interventions such as contact investigation, increased detection or to high DOTS compliance [19, 60, 61]. As has been previously described, we found that most of our genotype clusters were small [12, 30, 62, 63]. Yuen et. al. have recently proposed that small genotype clusters (less than five individuals) represent limited recent transmission, based on the hypothesis that populations that have limited TB transmission mostly due to effective TB control strategies differ from populations with uncontrolled transmission [64].

### Strengths and limitations of the study

We conducted a large molecular epidemiological study of pulmonary TB in an area where TB is endemic, and DM is increasing. Our study provided sociodemographic, clinical and epidemiological information that allowed us to obtain adjusted odds ratio associated with belonging to hotspots. Several studies have sought to determine the degree of correspondence between spatial aggregation and molecular clustering of patients with TB and test the hypothesis that both phenomena concur. While some authors have found that genotype-clustered cases share locations [28, 62, 65]; other authors have not confirmed this finding [29, 66]. A partial explanation for these discrepancies may be found in that multiple characteristics impact over genotype clustering frequency. These characteristics include among others, TB incidence, study duration, the intensity of contact tracing, migration patterns into the study area, size of molecular clusters, sampling fraction, the occurrence of endemic strains, the frequency of strains with low copy numbers, and age of study populations [67]. In the present study, we tried to deal with some aspects that might have limited our findings. We attempted to reduce the impact of the long duration of our study establishing that cases were identified within 12 months to be considered genotype-clustered. Throughout our study, we performed passive case finding supported by active case finding conducted by community-based health workers to decrease incomplete sampling. The results that there was no difference between the proportion of patients who received health care in the urban health center in Camerino Z. Mendoza between patients whose isolates were or were not genotyped indicates that the study was not biased regarding the probability of genotyping according to health centers. Therefore, we consider that genetic clustering is not explained by increased coverage of specific clinics. Migration into and out of the area may have limited our findings. Limitations include that although the cohort of patients was prospectively recruited, the present analysis was conducted retrospectively with the inherent problems of data quality that such design entails. Descriptive design of the study limits the possibility of determining if TB transmission occurred in the health centers. We were unable to culture and genotype all isolates. The main reason was the delay in receiving the sample in the laboratory due to the remoteness of the patient´s home and consequent low quality of the sample. We used strict criteria to define genetic clustering. Our definition might represent an underestimate of ‘true’ clustering. A definition which allowed nearly identical patterns might have included more isolates in the hotspot analysis. However, the impact on the hotspot analysis is more difficult to predict. Finally, since eligibility criteria for culturing differed along the study we compared the proportion of patients who underwent genotyping in each of the study periods; we found that proportion of genotyped patients were all above 75%. There were some differences when we compared patients whose isolates were fingerprinted with those among whom we were unable to fingerprint their isolates. This limitation might have affected the representativeness of our results to some subgroups such as individuals living in rural areas, particularly during 1995 to 1999.

## Conclusions

We successfully applied a combination of sociodemographic, clinic, molecular and geospatial analysis to study TB, providing evidence indicating the usefulness of these strategies for control programs of TB and DM in settings where both conditions are endemic. National TB programs emphasize the importance of timely detection, treatment and contact tracing. If transmission occurs more frequently in certain settings, further prevention and control measures might be implemented increasing their cost-effectiveness. For example, if our hypothesis on health centers proved correct, it would be necessary to adopt specific administrative, environmental, and personal protection measures in health units that provide care to patients with DM and patients with TB.

## Supporting information

S1 Fig Number of individuals at each stage of the study (1995–1999). (TIF)

S2 Fig Number of individuals at each stage of the study (2000–2005). (TIF)

S3 Fig Number of individuals at each stage of the study (2005–2010). (TIF)

S4 Fig Local spatial analysis of genotypically unclustered patients in the study area. Moran´s I revealed a pattern not significantly different than random. (Moran`s I = -0.001106, Z value = 0.085084, p = 0.9).
(TIF)

S1 Table Comparison of sociodemographic and clinical characteristics of patients with and without genotype information (1995–2010). (DOC)

S2 Table Comparison of sociodemographic and clinical characteristics of patients with and without genotype information (1995–1999). (DOCX)

S3 Table Comparison of sociodemographic and clinical characteristics of patients with and without genotype information (2000–2005). (DOCX)

S4 Table Comparison of sociodemographic and clinical characteristics of patients with and without genotype information (2005–2010). (DOCX)
