# Supplementary figures and images for: Genotyping and spatial analysis of pulmonary tuberculosis and diabetes cases in the state of Veracruz, Mexico
Source: PLoS One. 2018 Mar 13;13(3):e0193911. doi: 10.1371/journal.pone.0193911 (PMC5849303; doi:10.1371/journal.pone.0193911)

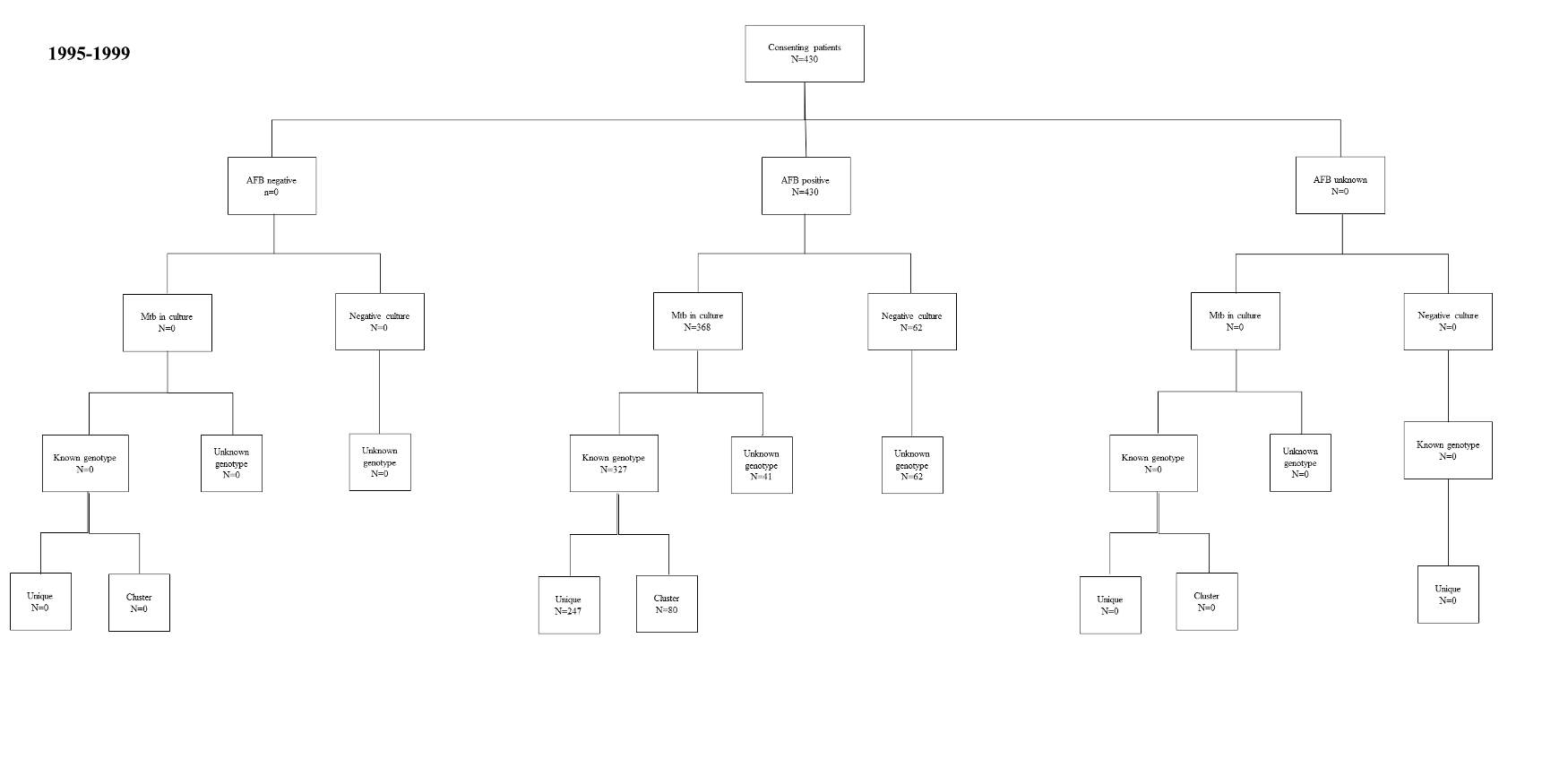

Supplement: S1 Fig — (TIF) [file pone.0193911.s001.tif]

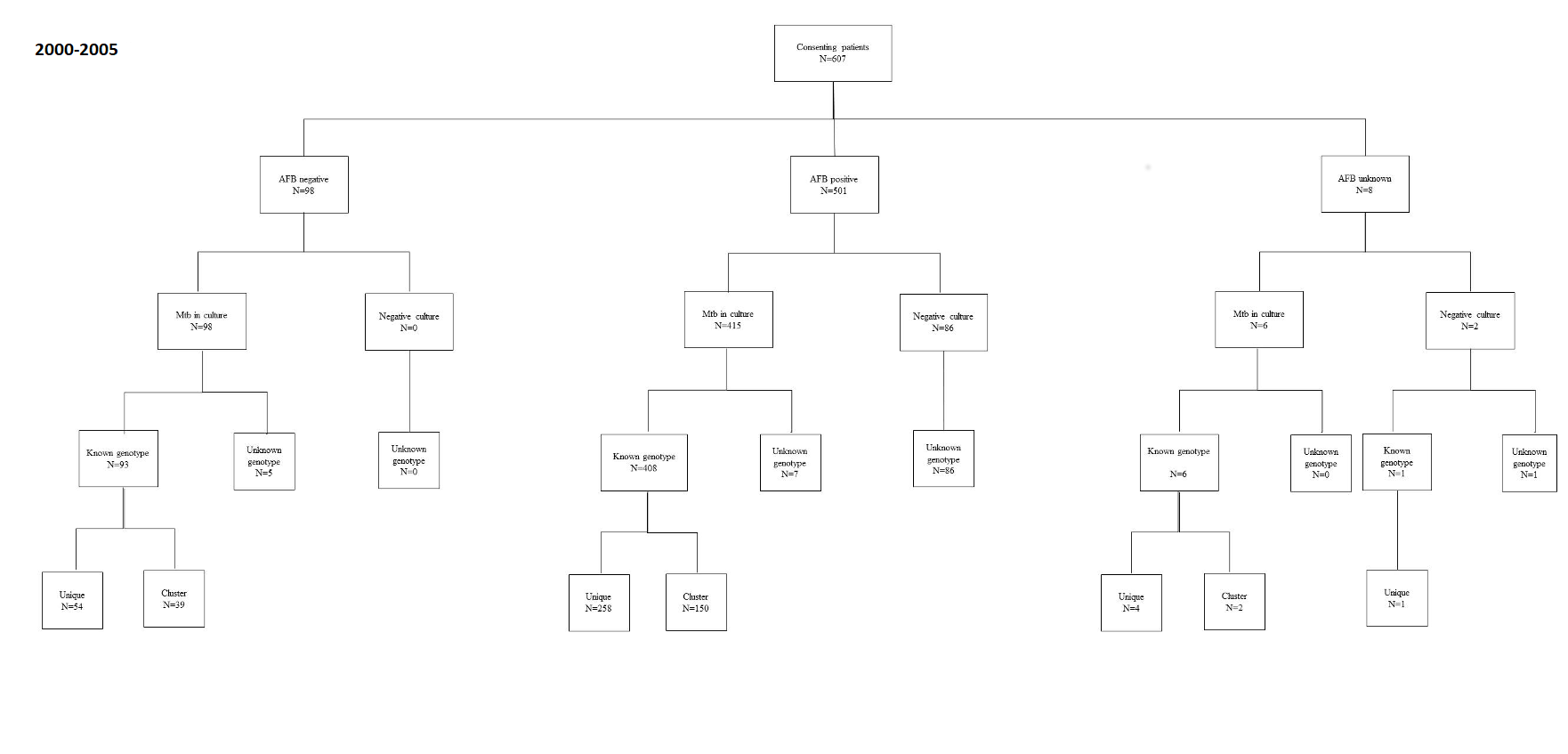

Supplement: S2 Fig — (TIF) [file pone.0193911.s002.tif]

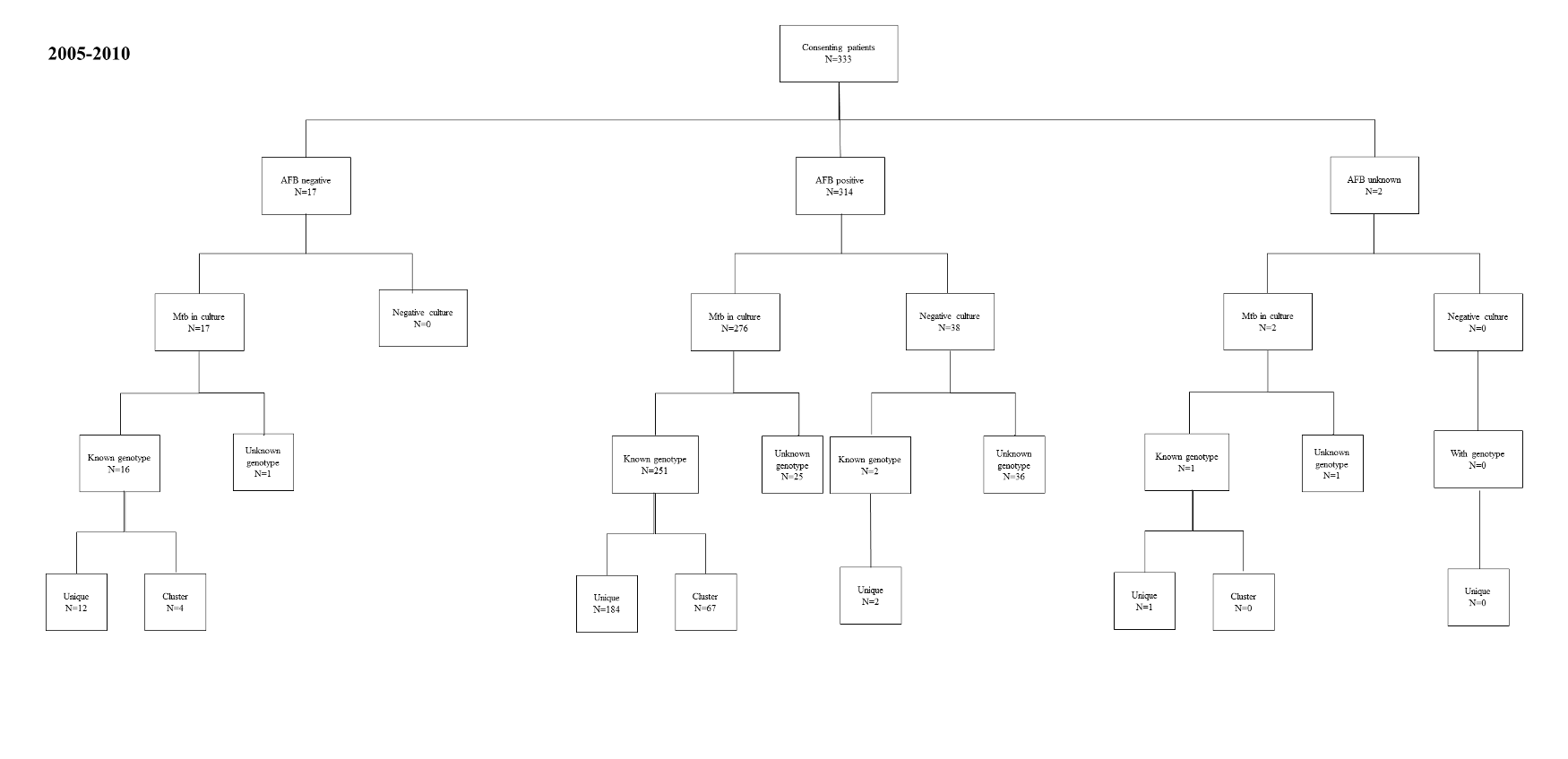

Supplement: S3 Fig — (TIF) [file pone.0193911.s003.tif]

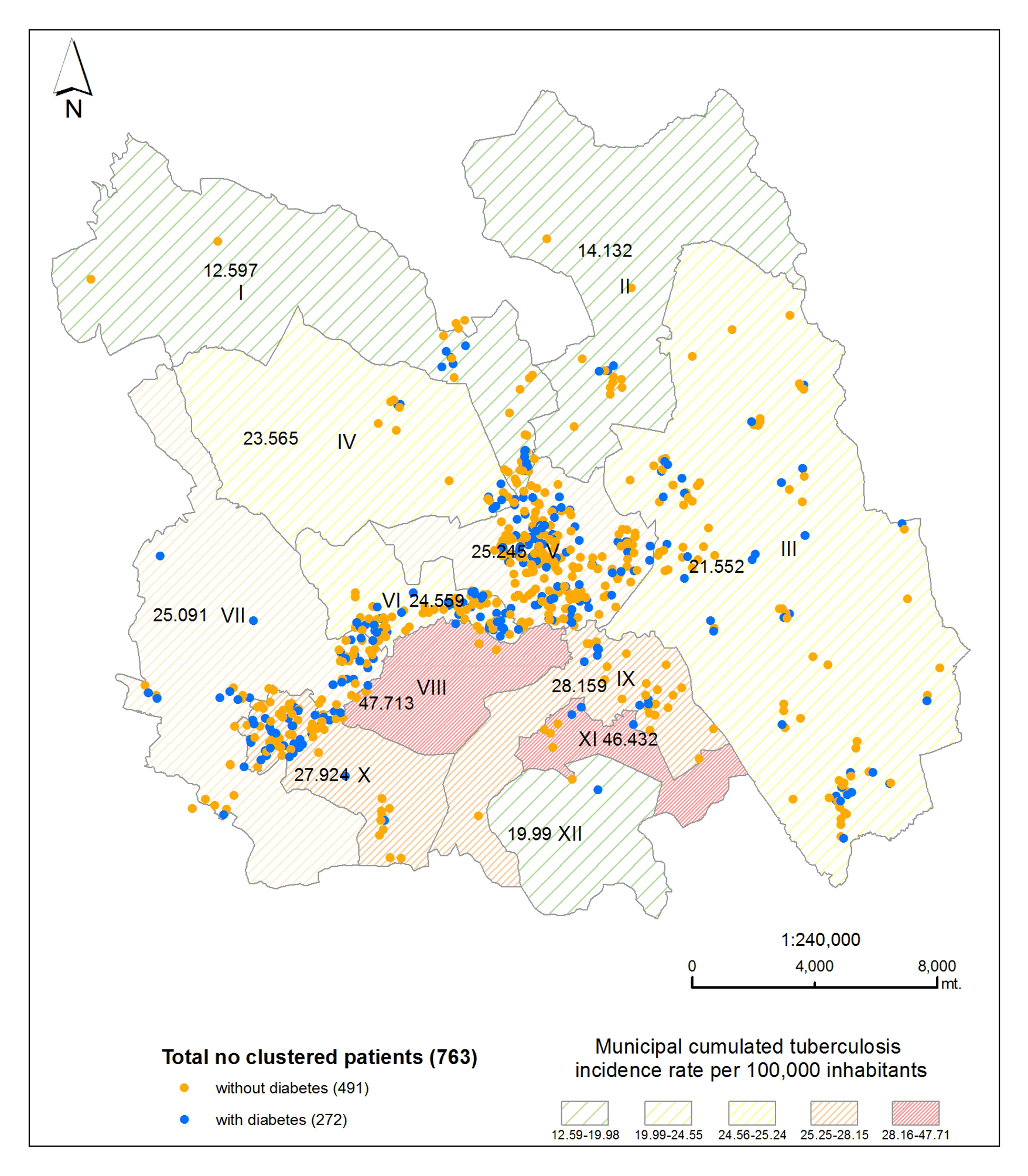

Supplement: S4 Fig — Moran´s I revealed a pattern not significantly different than random. (Moran`s I = -0.001106, Z value = 0.085084, p = 0.9). (TIF) [file pone.0193911.s004.tif]
