# Supplementary material for: Genotyping and spatial analysis of pulmonary tuberculosis and diabetes cases in the state of Veracruz, Mexico
Source: PLoS One. 2018 Mar 13;13(3):e0193911. doi: 10.1371/journal.pone.0193911 (PMC5849303; doi:10.1371/journal.pone.0193911)
Supplement: S1 Table — (DOC) [file pone.0193911.s005.doc]

# S1 Table . Comparison of sociodemographic and clinical characteristics of patients with and without RFLP information. Orizaba, Veracruz, 1995-2010.

| Characteristics | Total | Without RFLP-IS6110/ Spoligotype | With RFLP-IS6110/ Spoligotype | p-Value ^d^ |
| --- | --- | --- | --- | --- |
|  | n/total (%) | n/total (%) | n/total (%) |  |
| Male | 800/1370 (58.0) | 141/265 (53.0) | 659/1105 (60.0) | 0.056 |
| Mean (SD) age (years) | 45.5 (18.0) | 45.6 (19.5) | 45.5 (17.6) | 0.945^f^ |
| >6 years of formal schooling | 951/1369 (69.0) | 184/265 (69.0) | 767/1104 (69.0) | 0.990 |
| Household with earthen floor | 268/1370 (20.0) | 47/265 (18.0) | 221/1105 (20.0) | 0.404 |
| Rural residence | 148/1236 (12.0) | 30/244 (12.0) | 118/992 (12.0) | 0.863 |
| Median (IQR) distance to nearest health center (meters) | 701 (422-1045) | 744 (478-1173) | 696 (413-1034) | 0.161^e^ |
| Diagnosis between 2000 and 2010 | 940/1370 (69.0) | 162/265 (61.0) | 778/1105 (70.0) | 0.003 |
| Access to Social Security | 482/1370 (35.0) | 106/265 (40.0) | 376/1105 (34.0) | 0.067 |
| Urban health center in Camerino Z. Mendoza | 172/1368 (12.5) | 38/265 (14.3) | 134/1103 (12.1) | 0.334 ^d^ |
| Mean (SD) body mass index | 21.7 (6.0) | 23.5 (8.1) | 21.2 (5.3) | <0.001 ^f^ |
| >10 drinks per week | 595/1368 (43.0) | 101/265 (38.0) | 494/1103 (45.0) | 0.049 |
| >10 cigarettes per week | 310/1367 (23.0) | 49/264 (19.0) | 261/1103 (24.0) | 0.075 |
| Use of illegal drugs | 67/1369 (5.0) | 8/265 (3.0) | 59/1104 (5.0) | 0.115 |
| Homelessness or residing in shelters | 47/1365 (3.0) | 13/263 (5.0) | 34/1102 (3.0) | 0.138 |
| BCG scar ^a^ | 635/1362 (47.0) | 130/262 (50.0) | 505/1100 (46.0) | 0.279 |
| HIV infection ^b^ | 25/1322 (2.0) | 3/252 (1.0) | 22/1070 (2.0) | 0.364 |
| Median (IQR) time elapsed between onset of symptoms and treatment (days) | 105 (62-191) | 98 (52-188) | 107 (65.192) | 0.111^e^ |
| New tuberculosis patients | 1115/1369 (81.0) | 207/265 (78.0) | 908/1104 (82.0) | 0.120 |
| Diabetes Mellitus | 450/1370 (33.0) | 70/265 (26.0) | 380/1105 (34.0) | 0.013 |
| AFB in sputum |  |  |  |  |
| No bacilli in smear/M tuberculosis in culture | 115/1360 (8.0) | 6/263 (2.0) | 109/1097 (10.0) | <0.001 |
| 10 to 99 AFB^c^ per 100 immersion fields | 537/1360 (39.0) | 180/263 (68.0) | 357/1097 (33.0) |  |
| 1 to 10 AFB^c^ per oil immersion field | 385/1360 (28.0) | 53/263 (20.0) | 332/1097 (30.0) |  |
| More than 10 AFB^c^ per oil immersion field | 323/1360 (24.0) | 24/263 (9.0) | 299/1097 (27.0) |  |
| Drug susceptible | 781/1009 (77.0) | 42/64 (66.0) | 739/945 (78.0) | 0.020 |
| Multidrug resistant | 67/1009 (7.0) | 6/64 (9.0) | 61/945 (6.0) | 0.364 |
| Fever | 956/1367 (70.0) | 169/265 (64.0) | 787/1102 (71.0) | 0.015 |
| Haemoptysis | 450/1362 (33.0) | 84/261 (32.0) | 366/1101 (33.0) | 0.744 |
| Cavities on chest x-ray | 457/1155 (40.0) | 46/213 (22.0) | 411/942 (44.0) | <0.001 |

^a^BCG: vaccine against Bacillus Calmette-Guérin, ^b^HIV: human immunodeficiency virus, ^c^AFB: acid fast bacilli, ^d^ X^2^ test, ^e^Kruskall Wallis test, ^f^ Student's t-test.

SD, Standard deviation; IQR, Interquartile range.
