# Supplementary material for: Genotyping and spatial analysis of pulmonary tuberculosis and diabetes cases in the state of Veracruz, Mexico
Source: PLoS One. 2018 Mar 13;13(3):e0193911. doi: 10.1371/journal.pone.0193911 (PMC5849303; doi:10.1371/journal.pone.0193911)
Supplement: S2 Table — (DOCX) [file pone.0193911.s006.docx]

# S2 Table. Comparison of sociodemographic and clinical characteristics of patients with and without RFLP information. Orizaba, Veracruz, 1995-1999.

| Characteristics | Total | Without RFLP-IS6110/ Spoligotype | With RFLP-IS6110/ Spoligotype | p-Value ^d^ |
| --- | --- | --- | --- | --- |
|  | n/total (%) | n/total (%) | n/total (%) |  |
| Male | 253/430 (59.0) | 57/103 (55.0) | 196/327 (60.0) | 0.408 |
| Mean (SD) age (years) | 42.5 (17.4) | 41.7 (17.5) | 42.7 (17.4) | 0.618 ^f^ |
| >6 years of formal schooling | 286/429 (67.0) | 72/103 (70.0) | 214/326 (66.0) | 0.424 |
| Household with earthen floor | 67/430 (16.0) | 27/103 (26.0) | 40/327 (12.0) | 0.001 |
| Rural residence | 20/402 (5.0) | 9/99 (9.0) | 11/303 (4.0) | 0.03 |
| Median (IQR) distance to nearest health center (meters) | 710 (472-983) | 697 (512-1003) | 714 (471-983) | 0.738 |
| Access to Social Security | 154/430 (36.0) | 41/103 (40.0) | 113/327 (35.0) | 0.333 |
| Urban health center in Camerino Z. Mendoza | 64/430(14.8) | 19/103 (18.4) | 45/327 (13.7) | 0.244^d^ |
| Mean (SD) body mass index | 21.5 (18.0) | 23.0 (11.5) | 21.0 (7.2) | 0.023 ^f^ |
| >10 drinks per week | 197/429 (46.0) | 44/103 (43.0) | 153/326 (47.0) | 0.454 |
| >10 cigarettes per week | 132/429 (31.0) | 31/103 (30.0) | 101/326 (31.0) | 0.865 |
| Use of illegal drugs | 27/429 (6.0) | 5/103 (5.0) | 22/326 (7.0) | 0.49 |
| Homelessness or residing in shelters | 24/428 (6.0) | 8/103 (8.0) | 16/325 (5.0) | 0.274 |
| BCG scar ^a^ | 176/424 (42.0) | 38/100 (38.0) | 138/324 (43.0) | 0.415 |
| HIV infection ^b^ | 9/407 (2.0) | 1/93 (1.0) | 8/314 (3.0) | 0.396 |
| Median (IQR) time elapsed between onset of symptoms and treatment (days) | 109 (200-61) | 117 (65 -212) | 103 (61-197) | 0.399 |
| New tuberculosis patients | 324/430 (75.0) | 75/103 (73.0) | 249/327 (76.0) | 0.494 |
| Diabetes Mellitus | 132/430 (31.0) | 25/103 (24.0) | 107/327 (33.0) | 0.105 |
| AFB in sputum |  |  |  |  |
| No bacilli in smear/M tuberculosis in culture | 0/0 (0.0) | 0/0 (0.0) | 0/0 (0.0) | >0.001 |
| 10 to 99 AFB^c^ per 100 immersion fields | 201/430 (47.0) | 67/103 (65.0) | 134/327 (41.0) |  |
| 1 to 10 AFB^c^ per oil immersion field | 139/430 (32.0) | 27/103 (26.0) | 112/327 (34.0) |  |
| More than 10 AFB^c^ per oil immersion field | 90/430 (21.0) | 9/103 (9.0) | 81/327 (25.0) |  |
| Drug susceptible | 271/366 (74.0) | 28/40 (70.0) | 243/326 (75.0) | 0.536 |
| Multidrug resistant | 40/366 (11.0) | 4/40 (10.0) | 36/326 (11.0) | 0.842 |
| Fever | 353/430 (82.0) | 87/103 (84.0) | 266/327 (81.0) | 0.471 |
| Haemoptysis | 156/425 (37.0) | 42/100 (42.0) | 114/325 (35.0) | 0.209 |
| Cavities on chest x-ray | 143/399 (36.0) | 25/95 (26.0) | 118/304 (39.0) | 0.027 |

^a^BCG: vaccine against Bacillus Calmette-Guérin, ^b^HIV: human immunodeficiency virus, ^c^AFB: acid fast bacilli, ^d^ X^2^ test, ^e^Kruskall Wallis test, ^f^ Student's t-test.

SD, Standard deviation; IQR, Interquartile range.
