# Supplementary material for: Genotyping and spatial analysis of pulmonary tuberculosis and diabetes cases in the state of Veracruz, Mexico
Source: PLoS One. 2018 Mar 13;13(3):e0193911. doi: 10.1371/journal.pone.0193911 (PMC5849303; doi:10.1371/journal.pone.0193911)
Supplement: S3 Table — (DOCX) [file pone.0193911.s007.docx]

# S3 Table. Comparison of sociodemographic and clinical characteristics of patients with and without RFLP information. Orizaba, Veracruz, 2000-2005.

| Characteristics | Total | Without RFLP-IS6110/ Spoligotype | With RFLP-IS6110/ Spoligotype | p-Value ^d^ |
| --- | --- | --- | --- | --- |
|  | n/total (%) | n/total (%) | n/total (%) |  |
| Male | 346/607 (57.0) | 52/99 (53.0) | 294/508 (58.0) | 0.325 |
| Mean (SD) age (years) | 74.5 (18.3) | 51.2 (20.8) | 46.8 (17.7) | 0.041 ^f^ |
| >6 years of formal schooling | 454/607 (75.0) | 72/99 (73.0) | 382/508 (75.0) | 0.605 |
| Household with earthen floor | 138/607 (23.0) | 12/99 (12.0) | 126/508 (25.0) | 0.006 |
| Rural residence | 75/543 (14.0) | 9/89 (10.0) | 66/454 (15.0) | 0.269 |
| Median (IQR) distance to nearest health center (meters) | 701 (424-1103) | 768 (517-1148) | 694 (413-1095) | 0.186 |
| Access to Social Security | 222/607 (37.0) | 44/99 (44.0) | 178/508 (35.0) | 0.075 |
| Urban health center in Camerino Z. Mendoza | 78/606 (12.8) | 17/99 (17.1) | 61/507 (12.0) | 0.162^d^ |
| Mean (SD) body mass index | 21.8 (4.6) | 24.9 (4.9) | 21.2 (4.3) | <0.001 ^f^ |
| >10 drinks per week | 254/607 (42.0) | 32/99 (32.0) | 222/508 (44.0) | 0.036 |
| >10 cigarettes per week | 116/607 (19.0) | 7/99 (7.0) | 109/508 (21.0) | 0.001 |
| Use of illegal drugs | 31/607 (5.0) | 1/99 (1.0) | 30/508 (6.0) | 0.043 |
| Homelessness or residing in shelters | 13/607 (2.0) | 3/99 (3.0) | 10/508 (2.0) | 0.504 |
| BCG scar ^a^ | 274/606 (45.0) | 54/99 (55.0) | 220/507 (43.0) | 0.041 |
| HIV infection ^b^ | 11/594 (2.0) | 1/99 (1.0) | 10/495 (2.0) | 0.496 |
| Median (IQR) time elapsed between onset of symptoms and treatment (days) | 105 (63-182) | 75 (39-125) | 112 (67-186) | <0.001 |
| New tuberculosis patients | 502/607 (83.0) | 80/99 (81.0) | 422/508 (83.0) | 0.586 |
| Diabetes Mellitus | 204/607 (34.0) | 30/99 (30.0) | 174/508 (34.0) | 0.447 |
| AFB in sputum |  |  |  |  |
| No bacilli in smear/M tuberculosis in culture | 98/599 (16.0) | 5/98 (5.0) | 93/501 (19.0) | <0.001 |
| 10 to 99 AFB^c^ per 100 immersion fields | 230/599 (38.0) | 78/98 (80.0) | 152/501 (30.0) |  |
| 1 to 10 AFB^c^ per oil immersion field | 149/599 (25.0) | 12/98 (12.0) | 137/501 (27.0) |  |
| More than 10 AFB^c^ per oil immersion field | 122/599 (20.0) | 3/98 (3.0) | 119/501 (24.0) |  |
| Drug susceptible | 415/508 (82.0) | 7/11 (64.0) | 408/497 (82.0) | 0.117 |
| Multidrug resistant | 18/508 (4.0) | 1/11 (9.0) | 17/497 (3.0) | 0.314 |
| Fever | 377/604 (62.0) | 40/99 (40.0) | 337/505 (67.0) | <0.001 |
| Haemoptysis | 188/605 (31.0) | 23/99 (23.0) | 165/506 (33.0) | 0.065 |
| Cavities on chest x-ray | 195/547 (36.0) | 9/91 (10.0) | 186/456 (41.0) | <0.001 |

^a^BCG: vaccine against Bacillus Calmette-Guérin, ^b^HIV: human immunodeficiency virus, ^c^AFB: acid fast bacilli, ^d^ X^2^ test, ^e^Kruskall Wallis test, ^f^ Student's t-test.

SD, Standard deviation; IQR, Interquartile range.
