# Supplementary material for: Genotyping and spatial analysis of pulmonary tuberculosis and diabetes cases in the state of Veracruz, Mexico
Source: PLoS One. 2018 Mar 13;13(3):e0193911. doi: 10.1371/journal.pone.0193911 (PMC5849303; doi:10.1371/journal.pone.0193911)
Supplement: S4 Table — (DOCX) [file pone.0193911.s008.docx]

# S4 Table. Comparison of sociodemographic and clinical characteristics of patients with and without RFLP information. Orizaba, Veracruz, 2005- 2010.

| Characteristics | Total | Without RFLP-IS6110/ Spoligotype | With RFLP-IS6110/ Spoligotype | p-Value ^d^ |
| --- | --- | --- | --- | --- |
|  | n/total (%) | n/total (%) | n/total (%) |  |
| Male | 201/333 (60.0) | 32/63 (51.0) | 169/270 (63.0) | 0.085 |
| Mean (SD) age (years) | 45.6 (17.8) | 43.0 (18.9) | 46.3 (17.5) | 0.219 ^f^ |
| >6 years of formal schooling | 211/333 (63.0) | 40/63 (63.0) | 171/270 (63.0) | 0.981 |
| Household with earthen floor | 63/333 (19.0) | 8/63 (13.0) | 55/270 (20.0) | 0.162 |
| Rural residence | 53/291 (18.0) | 12/56 (21.0) | 41/235 (17.0) | 0.488 |
| Median (IQR) distance to nearest health center (meters) | 674 (339-1085) | 778 (319-1198) | 667 (348-1053) | 0.483 |
| Access to Social Security | 106/333 (32.0) | 21/63 (33.0) | 85/270 (31.0) | 0.776 |
| Urban health center in Camerino Z. Mendoza | 30/332 (9.0) | 2/63 (3.1) | 28/269 (9.04) | 0.071^d^ |
| Mean (SD) body mass index | 21.5 (3.9) | 21.7 (4.1) | 21.4 (3.9) | 0.503 ^f^ |
| >10 drinks per week | 144/332 (43.0) | 25/63 (40.0) | 119/269 (44.0) | 0.511 |
| >10 cigarettes per week | 62/331 (19.0) | 11/62 (18.0) | 51/269 (19.0) | 0.825 |
| Use of illegal drugs | 9/333 (3.0) | 2/63 (3.0) | 7/270 (3.0) | 0.798 |
| Homelessness or residing in shelters | 10/330 (3.0) | 2/61 (3.0) | 8/269 (3.0) | 0.900 |
| BCG scar ^a^ | 185/332 (56.0) | 38/63 (60.0) | 147/269 (55.0) | 0.415 |
| HIV infection ^b^ | 5/321 (2.0) | 1/60 (2.0) | 4/261 (2.0) | 0.94 |
| Median (IQR) time elapsed between onset of symptoms and treatment (days) | 104 (64-199) | 63 (110-217) | 64 (103-195) | 0.961 |
| New tuberculosis patients | 289/332 (87.0) | 52/63 (83.0) | 237/269 (88.0) | 0.236 |
| Diabetes Mellitus | 114/333 (34.0) | 15/63 (24.0) | 99/270 (37.0) | 0.053 |
| AFB in sputum |  |  |  |  |
| No bacilli in smear/M tuberculosis in culture | 17/331 (5.0) | 1/62 (2.0) | 16/269 (6.0) | <0.001 |
| 10 to 99 AFB^c^ per 100 immersion fields | 106/331 (32.0) | 35/62 (56.0) | 71/269 (26.0) |  |
| 1 to 10 AFB^c^ per oil immersion field | 97/331 (29.0) | 14/62 (23.0) | 83/269 (31.0) |  |
| More than 10 AFB^c^ per oil immersion field | 111/331 (34.0) | 12/62 (19.0) | 99/269 (37.0) |  |
| Drug susceptible | 95/135 (70.0) | 7/13 (54.0) | 88/122 (72.0) | 0.17 |
| Multidrug resistant | 9/135 (7.0) | 1/13 (8.0) | 8/122 (7.0) | 0.876 |
| Fever | 226/333 (68.0) | 42/63 (67.0) | 184/270 (68.0) | 0.821 |
| Haemoptysis | 106/332 (32.0) | 19/62 (31.0) | 87/270 (32.0) | 0.81 |
| Cavities on chest x-ray | 119/209 (57.0) | 12/27 (44.0) | 107/182 (59.0) | 0.16 |

^a^BCG: vaccine against Bacillus Calmette-Guérin, ^b^HIV: human immunodeficiency virus, ^c^AFB: acid fast bacilli, ^d^ X^2^ test, ^e^Kruskall Wallis test, ^f^ Student's t-test.

SD, Standard deviation; IQR, Interquartile range.
